# Obtaining and Characterization of Biodegradable Polymer Blends Based on Polyvinyl Alcohol, Starch, and Chitosan

**DOI:** 10.3390/polym17040479

**Published:** 2025-02-12

**Authors:** Galiya Irmukhametova, Khaldun M. Al Azzam, Grigoriy A. Mun, Lyazzat Bekbayeva, Zhetpisbay Dinara, Bayana B. Yermukhambetova, Sergey V. Nechipurenko, Sergey A. Efremov, El-Sayed Negim, Moshera Samy

**Affiliations:** 1 Department of Chemistry & Technology of Organic Materials, Polymers and Natural Compounds, Al-Farabi Kazakh National University, 71 Al-Farabi Av., Almaty 050040, Kazakhstan; mungrig@yandex.ru (G.A.M.); sergey.nechipurenko@kaznu.edu.kz (S.V.N.); sergey.efremov@kaznu.edu.kz (S.A.E.); 2 National Engineering Academy of the Republic of Kazakhstan, 80 Bogenbai Batyr Str., Almaty 050010, Kazakhstan; lyazzat_bk2019@mail.ru (L.B.); zhetpisbay.d@kaznmu.kz (Z.D.); baya_yerm@mail.ru (B.B.Y.); 3 Department of Chemistry, Faculty of Science, The University of Jordan, Amman 11942, Jordan; azzamkha@yahoo.com; 4 National Nanotechnology Open Laboratory, Al-Farabi Kazakh National University, 71 Al-Farabi Av., Almaty 050040, Kazakhstan; 5 Department of Biochemistry, School of General Medicine-1, KazNMU Named S.D. Asfendiyarov, 88 Tole bi Str., Almaty 480012, Kazakhstan; 6 School of Materials Science and Green Technologies, Kazakh British Technical University, 59 Tole bi Str., Almaty 050000, Kazakhstan; elashmawi5@yahoo.com; 7 Polymers and Pigments Department, National Research Centre, 33 El Buhouth St., Dokki, Giza 12622, Egypt; moshera_samy1984@yahoo.com

**Keywords:** chitosan, polyvinyl alcohol, biodegradable, mechanical properties

## Abstract

Although chitosan (CS) is used in many industries because of its low cost, biodegradability, nontoxic, antibacterial, and antioxidant qualities, it lacks sufficient mechanical and barrier properties. Biodegradable polymers based on CS, polyvinyl alcohol (PVA), and starch (S) were prepared at various ratios (1/3/6 and 1/5/4) via a blending polymerization process in the presence of water as the solvent and glacial acetic acid as the catalyst. The obtained biodegradable polymers were characterized via FTIR, TGA, SEM, and mechanical tests. The biodegradable polymers were mixed with rice straw and carbon black to study the effects of rice straw and carbon black on the physicomechanical properties of the biodegradable polymer films, including viscosity, tensile strength, elongation, and contact angle. The incorporation of rice straw and carbon black into a polymer blend significantly enhanced the physical and mechanical properties while also boosting their biodegradability by 36% and 15%, respectively, due to their biological activity. Notably, the CS/PVA/S blend with a ratio of 1/5/4, combined with rice straw, emerged as the standout performer. It exhibited superior mechanical strength and the shortest degradation time, outperforming the CS/PVA/S blended with a ratio of 1/3/6 mixed with carbon black. According to these findings, the biodegradable polymers became more soluble as the temperature increased from 30 to 45 °C.

## 1. Introduction

In recent years, biodegradable polymers have garnered attention because of their ability to degrade into nontoxic and environmentally friendly materials [1]. Mechanical strength, thermal, and electrical properties of common biodegradable polymers and their blending [2]. Chitosan (CS), a biodegradable polymer, is a deacetylated derivative of natural chitin that is primarily composed of 1→4 linked 2-amino-2-deoxy-β-D-glucopyranose (D-glucosamine) units [3]. CS is regarded as the most promising polysaccharide for biomedical applications [4]. In general, CS is nontoxic, antibacterial, biocompatible, and biodegradable. CS has several uses in the food, agricultural, medical, and cosmetics industries because of its strong antioxidant, antibacterial, biodegradability, nontoxicity, availability, low cost [5], and advantageous physicochemical and biological characteristics [6]. Despite its beneficial features, it lacks adequate mechanical and barrier properties [6]. Many chemical or physical CS modifications, including blending, crosslinking, chemical alterations, complexation, and graft copolymerization [7], have been studied to increase the quality of CS and expand its range of applications [6,8]. Specifically, adding new, desirable features to CS through mixing is an appealing modification technique that has been widely employed. It is easy to use, useful for real-world applications, and readily available in various synthetic and natural polymers for blending [9,10]. The inadequate mechanical properties of CS materials limit their use in biomedicine [4]. This can be solved by adding biodegradable polymers such as those with CS, such as the biopolymers starch (S) and polyvinyl alcohol (PVA), which have good mechanical properties, to CS. The cationic nature of CS causes some linkages between its amine groups and the hydroxyl groups of PVA. PVA is a water-soluble synthetic polymer produced by the hydrolysis of polyvinyl acetate, making it an ideal packaging material.

PVA is a synthetic, semicrystalline, white material that is used as a crosslinker for the hydrogel support matrix because of its high mechanical strength, hydraulic shock resistance, hydrophilicity, nontoxicity, and low cost. It is a long-chain water-soluble polymer that increases hydrophobicity and stability, making it suitable for various applications. PVA-based hydrogels are used in the food and biomedical industries for medication carriers, tissue engineering scaffolds, artificial muscles, wound dressings, and biosensors. To enhance the mechanical properties of hydrogels, PVA is often combined with other materials to create hydrogels [11].

The blending of CS with S, a natural biopolymer, is due to its abundance, cost-effectiveness, biodegradability, and nonimmunogenic properties, as well as its potential use in the manufacture of biodegradable films in the form of thermoplastic starch (TPS). Nevertheless, the TPS film is constrained by its hydrophilic characteristics, which lead to alterations in its mechanical properties upon exposure to elevated relative humidity. Therefore, the incorporation of CS is proposed to mitigate this constraint. The CS/S blend has excellent film-forming characteristics due to the intra- and intermolecular hydrogen bonds formed between the hydroxyl and amino groups on the backbones of the two components [12]. The CS-to-S ratio affects the mechanical, water barrier, and miscibility characteristics of biodegradable mixed films [13]. Compared with the individual components, the resulting blends exhibit increased hydrophilicity, mechanical qualities, and biocompatibility. PVA is a synthetic hydrophilic polymer with exceptional characteristics due to the formation of hydrogen bonds and the prevalence of –OH groups [14,15]. It is distinguished by elevated dielectric strength, good charge storage capacity, and superior mechanical qualities. PVA exhibits remarkable compatibility with CS, possesses adhesive qualities, and demonstrates film-forming capabilities through casting and great chemical resistance to organic solvents [16]. PVA is used in various applications, including packaging, polymer recycling, membrane preparation, and controlled drug delivery systems [17]. Despite being a biodegradable polymer, PVA has no biological activity. The incorporation of the naturally occurring polysaccharide CS may stimulate the antibacterial activity of PVA. Blending CS with PVA is a desirable technique to create biodegradable polymers with improved qualities, such as enhanced mechanical and water retention [18,19]. Compared with their polymers, the resulting mixtures are novel materials with improved mechanical, hydrophilic, and biocompatible qualities [20]. Finally, the blend of CS with S and PVA is expected to exhibit good mechanical and process properties [21]. Owing to the strong interaction among the hydroxyl groups on S chains and PVA, the excellent compatibility of these biodegradable polymers results in better mechanical properties than the characteristics of a single biodegradable polymer [22,23].

A comprehensive literature review reveals limited reports on the blending of PVA and CS without the incorporation of S. Notable studies include the preparation and characterization of PVA/CS blends plasticized and compatibilized with glycerol/polyethylene glycol, as well as the fabrication and characterization of films based on poly(vinyl alcohol) and CS oligosaccharides [24,25,26]. Additionally, investigations have investigated active films composed of poly(vinyl alcohol) and CS/poly(vinyl alcohol) infused with white turmeric powder for food packaging purposes [27], and combinations of CS, polyethylene glycol, polyvinyl alcohol, and polyvinylpyrrolidone [28]. In all these instances, the combination of PVA, CS, and S for creating biodegradable polymer blends, including polyvinyl alcohol, S, and CS, has not been utilized.

This study examines the obtaining and structural features of biodegradable polymer blends composed of CS, S, and polyvinyl alcohol, focusing on their mechanical, structural, and biological qualities. Previous literature reviews have examined the blending of PVA, CS, and S individually with other materials; however, our study presents a novel approach involving blending PVA, CS, and S. This work provides insights into synthesizing and characterizing these materials for various human contact applications. Furthermore, to improve the biodegradability of the obtained films, blended polymer mixtures of rice straw and carbon black, and characterize the structure of the prepared films, Fourier-transform infrared (FTIR) spectroscopy, thermogravimetric analysis (TGA), and scanning electron microscopy (SEM) were also performed.

## 2. Materials and Methods

CS with a low molecular weight (50,000–190,000 Da (based on viscosity)) and degree of deacetylation ≥75% and glacial acetic acid was purchased from Sigma-Aldrich Company (St. Louis, MO, USA). Deacetylation was confirmed via FTIR analysis (Supplementary File S1). Corn starch (S, Mw 692.65 g/mol) was obtained from “Everest” Ltd. (Moscow, Russia). PVA with a molecular weight of 70,000–100,000 g/mol was purchased from Sigma-Aldrich Company (St. Louis, MO, USA). Rice straw was collected from the fields and air-dried as previously described [29]. The rice straw was subsequently cut into 1–3 mm long pieces and milled to powder. Carbon black was purchased from Balausa Firm LLP (Kyzylorda, Kazakhstan), where its carbon content is 39.5%; the particle size (−10 microns) is 90%; the bulk density is 430 kg/m^3^; the specific adsorption surface area is 79.9 m^2^/g; the absorption of dibutyl phthalate is 90 cm^3^/100 g; and the humidity is 0.5%. X-ray spectral analysis of a black carbon sample revealed the following results: Na_2_O: 0.40%; MgO: 0.34%; Al_2_O_3_: 7.74%; SiO_2_: 40.36%; P_2_O_5_: 0.60%; K_2_O: 1.66%; CaO: 1.30%; TiO_2_: 0.37%; MnO: <0.10%; V_2_O_5_: 0.44%; BaO: 0.48%; SO_3_: 2.33%; Fe_2_O_3_: 2.45%; and p.p.p: 41.53%.

### 2.1. Obtaining of Chitosan-b-Polyvinyl Alcohol-b-Starch (CS-b-PVA)

**Figure 1:**
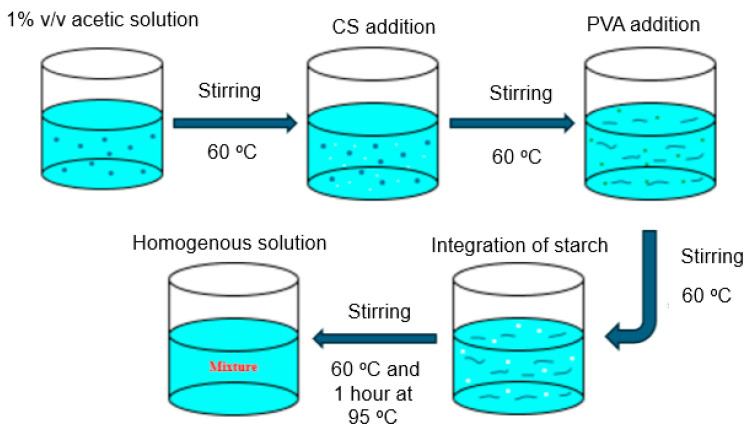
Schematic diagram of the preparation of the blending polymerization mixture of chitosan-b-polyvinyl alcohol-b-starch (CS-b-PVA).

Blending polymerization was carried out in a three-necked flask under a nitrogen atmosphere with a mechanical stirrer. A total of 1.0 g of CS was dissolved in 1% *v*/*v* acetic acid and continuously agitated at 60 °C (Figure 1). In brief, a 1% acetic acid solution was prepared and maintained at 60 °C. To achieve adequate mixing, CS is added to this solution while it is constantly stirred. After that, PVA was added gradually to the mixture while the temperature was maintained at 60 °C so that it dissolved uniformly. S was then added to the mixture at 60 °C while it was being mixed. To encourage more interactions, the mixture was then heated for an hour at 95 °C after being swirled and heated to 60 °C at first. To create a homogenous and consistent mixture, constant stirring was used. Prior literature reviews have investigated the individual mixing of PVA, CS, and S with various materials; however, our work introduces an innovative method by concurrently blending PVA, CS, and S. Upon complete dissolution of the CS, a freshly prepared aqueous solution of PVA, 3 g or 6 g) and 50 mL distilled water was added to the flask which was heated at 60 °C until a homogenous mixture was obtained. Subsequently, 6 or 4 g of S were dissolved in 100 mL of distilled water, introduced into three-necked flasks, and heated at 60 °C for 1 h. After that, the reaction was performed for another two hours while stirring at 500 rpm at 95 °C, followed by an additional 20 min at room temperature. Two different ratios of PVA and S were used to study the effects of the concentrations of PVA and S on the mechanical and biodegradability properties of the films in the presence of CS. The specifications of the mixed polymers are listed in Table 1.

### 2.2. Film Formation

The casting solution method produced CS/PVA/S films in a thoroughly cleaned glass dish. The fabrication of the CS/PVA/S films involved depositing their aqueous solutions onto flat glass surfaces and allowing them to dry at ambient temperature (25 °C) with a relative humidity of 59% for 7 days, followed by drying in an aerated oven at 60 °C for 12 h, after which the films were weighed more than 3 times until a constant value was reached to ensure complete water removal [30,31]. The films were subsequently dried and stored at ambient temperature in a desiccator for further characterization and measurements. CS/PVA/S at a ratio of 1/3/6 combined with 10% carbon black is designated M3, whereas CS/PVA/S at a ratio of 1/5/4 mixed with 10% rice straw is designated M4. Note that we fixed the amounts of CS, rice straw, and carbon black to investigate the impact of the PVA and S concentrations on the characteristics of the films. At the same time, we kept the amounts of carbon black and rice straw constant. Additionally, 10% is the greatest amount of waste material that can be used.

### 2.3. Measurements

FTIR absorbance spectra were acquired via a Bruker Tensor 37 FTIR spectrometer. The spectra were acquired from 4000 to 400 cm^−1^. The viscosity (η) of the dispersions was assessed using a Brookfield viscometer (Model LVTDV-II) at a shear rate of 100 s^−1^ at 25 °C. The contact angle between the water droplets and the sample surface was measured via a CAHN DCA-322 contact angle measurement device, operating at 25 °C with a water droplet and a velocity of 100 µm/s. A droplet of water was positioned on the surface for testing using a microsyringe, and the contact angle was determined from the perspective of the water droplets displayed on the monitor (three measurements taken from different areas of the film). Before the FTIR measurements, the polymers were dehydrated at 35 °C for 4–5 h and positioned in a humidity chamber until analysis. TGA was conducted with a TGA/SDTA851e apparatus from METTLER TOLEDO. The studies were performed at a heating rate of 10 °C per minute throughout the 30 to 900 °C temperature range. The TGA data indicate the thermostability and degradation temperature. The tensile strength and elongation at the break of the film were assessed according to ASTM D 882-91 [32], which uses an MTS 10/M tensile testing apparatus at a 50 mm/min crosshead speed. The tensile strength and elongation at break were assessed, with a minimum of three measurements averaged, utilizing a 1-kN load cell. The thermostability and temperature of degradation were assessed with TGA. Experiments were conducted on film biodegradation (three samples for each film) using regular soil within a plastic container. The degradation of the films was investigated using methods reported by the authors [33,34]. Experiments were conducted on film biodegradation using regular soil within a plastic container. Two types of soils were utilized, one from the Al-Faraby Kazakh National University courtyard in Almaty, Kazakhstan, and the other dry. After being buried for 80 days in dry and moist soils, the biodegradability of the polymeric films was assessed. The degree of film solubility was evaluated by measuring the content of dry matter solubilized after immersion in water at various temperatures (30, 35, 40, and 45 °C) and comparing the results to the dry weight before immersion. The degree of solubility of the films was calculated using the following formula:

% Solubility = [(m − m_0_)/m] × 100

where m and m_0_ represent the weights of the films before and after immersion, respectively.

## 3. Results and Discussion

### 3.1. FTIR Spectra

**Figure 2:**
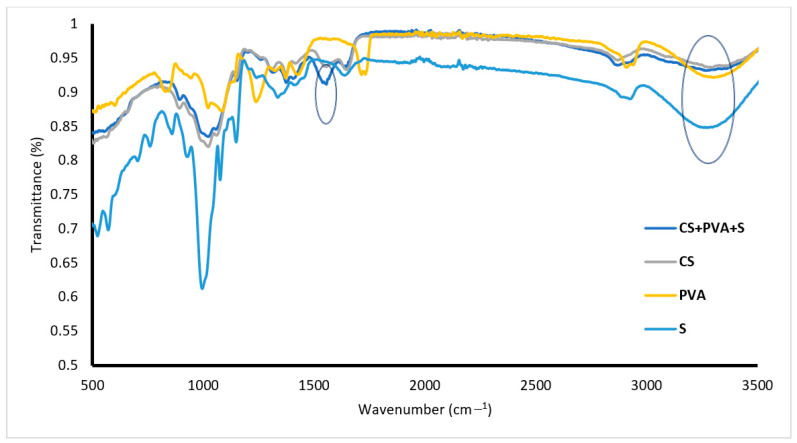
FTIR spectra of PVA, S, CS and CS/PVA/S.

Figure 2 shows the FTIR spectrum of PVA, with a peak at 2737 cm^−1^ corresponding to CH_2_ asymmetric stretching, a peak at 3400 cm^−1^ related to OH, and a peak at 1040 cm^−1^ corresponding to C–O stretching. The FTIR spectra of S are presented in Figure 2, whereas the absorption peaks at 3250, 2800, 1200, and 1000–1050 cm^−1^ correspond to OH, C–H, C–O–C, and C–O, respectively. FTIR spectrum of CS showed that peaks at 1162, 1080, 1028, and 970 cm^−1^ were attributed to OH (bending), C–O (stretching), and C–N (stretching), respectively. The peak at 3350 cm^−1^ is related to the stretching vibrations of O–H and N–H. The CS/PVA/S spectrum (Figure 2) indicates that new peaks appear at 1580 cm^−1^ for N–H (stretching) and at 1450 cm^−1^ and 1320 cm^−1^ for CH_2_ (bending) and CH_3_ (symmetrical) from CS due to the blending process [18]. As shown in Figure 2, the transmittance of the hydroxy group decreased, which was attributed to the blending of PVA with CS [35]. The FTIR spectra of both CS and S are similar because of the cyclic structures and many –OH bonds characteristic of saccharides, water, and carboxylic acids in both CS and S. The latter is supported by the work of Ngwabebhoh et al., 2016 [36] and of Lozano-Navarro et al., 2018 [37].

### 3.2. Viscosity

**Figure 3:**
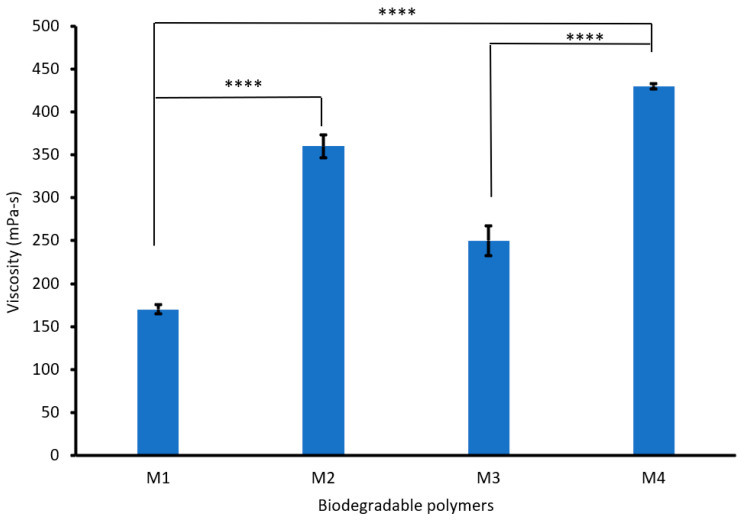
Viscosity values for the biodegradable polymers mixed with rice straw and carbon black. A statistically significant difference is given as ****—*p* < 0.0001 and no statistically significant difference—*p* > 0.05.

Figure 3 shows that increasing the PVA content from 3 to 6% increased the viscosity of the biodegradable polymers (CS/PVA/S) from 170 to 360 mPa-s. This increase in viscosity is due to the flexibility and hydrogen bonds between PVA, CS, and S [38]. However, mixing CS/PVA/S at a ratio of 1/3/6 with carbon black (M3) increased the viscosity of the biodegradable polymer from 170 mPa-s to 250 mPa-s, whereas mixing CS/PVA/S at a ratio of 1/5/4 (M4) increased the viscosity of the biodegradable polymer from 360 mPa-s to 430 mPa-s. Generally, the viscosity of the polymer is influenced by different factors, including temperature, additives, the structure of the polymer, and functional groups [39].

### 3.3. Thermogravimetric Analysis (TGA)

**Figure 4:**
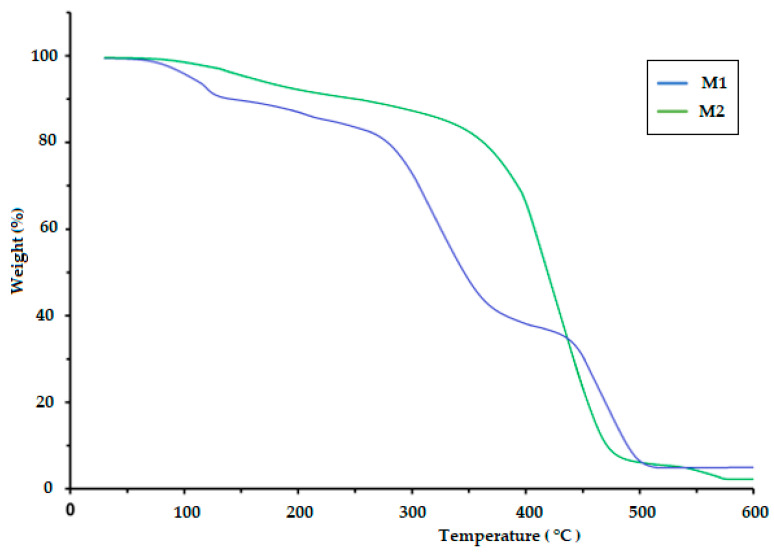
TGA thermogram of the biodegradable polymers CS/PVA/S (M1 and M2).

**Figure 5:**
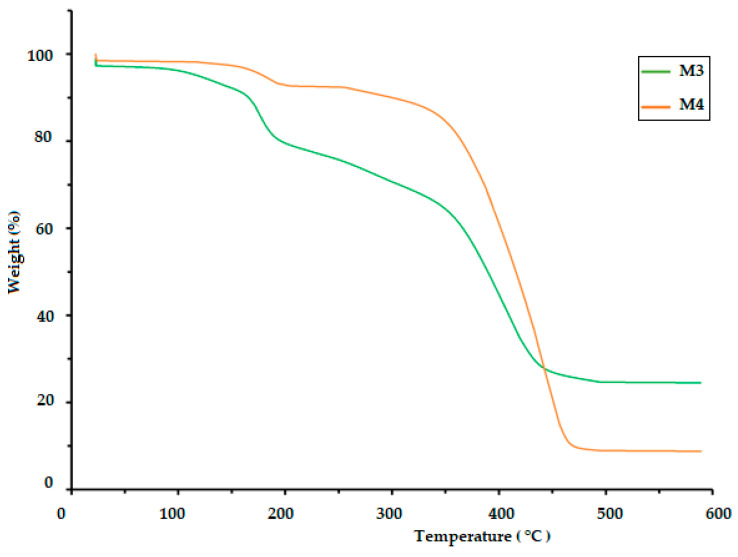
TGA thermogram of the biodegradable polymers CS/PVA/S mixed with rice straw and carbon black (M3 and M4).

The TGA of polymeric materials is related to measuring weight loss as a function of temperature and time. This is attributed to the influence of temperature on the structure and weight of the polymer, which is caused by decomposition and oxidation reactions. The TGA results for the CS/PVA/S biopolymer at ratios of 1/6/3 (M1) and 1/5/4 (M2) are presented in Figure 4 and Table 2. The TGA results were evaluated at temperatures ranging from 0.0 C to 900 °C. The thermal decomposition process of M1 proceeded in four stages, but the thermal decomposition process of M2 proceeded in two stages, as shown in Table 2. The first stage for M1 was at a temperature of 200 °C, and the weight loss was 20%, whereas the first stage for M2 was at a temperature of 420 °C, and weight loss was 18% due to the loss of water and moisture [40]. The second stage of M1 occurred at temperatures in the range of 280 to 340 °C with a weight loss of 41%, but the weight loss percentage for the third stage was 18% at temperatures ranging from 340 to 470 °C due to the decomposition of S. The fourth stage for M1 occurred at temperatures in the range of 470–500 °C, and the percentage weight loss was 25%, whereas the second stage for M2 occurred at temperatures in the range of 420–500 °C with a weight loss of 75% due to the decomposition of polyvinyl alcohol, CS and S [40,41]. Table 2 and Figure 5 show the thermal decomposition of CS/PV/S at a ratio of 1/3/6 mixed with black color (M3) and the thermal decomposition of CS/PV/S mixtures with ratios of 1/5/4 mixed with rice straw (M4). The thermal decomposition of M3 proceeded in four stages, whereas that of M4 occurred in two stages. The first stage for M3 occurred at 200 °C with a weight loss of 20%, whereas the first stage for M4 occurred at 400 °C with a weight loss of 20%, because of the thermal stability of M4 in the presence of rice straw. The weight loss is attributed to the removal of water and moisture [40]. The second and third stages for M3 were at temperatures in the range of 200–470, with a weight loss of 25% for the second stage and 40% for the third stage due to the loss of carbon black and S. The weight loss percentage for M3 was 15%, whereas for M4, it was 60% at temperatures 470–600 for M3 and 400–500. This is attributed to the loss of CS, PVA, S, and rice straw [42,43,44,45,46].

### 3.4. Mechanical Properties

Preliminary results provided a detailed comparison of the mechanical properties and biodegradability of films made from CS/PVA/S with two different additives (carbon black and rice straw) at varying concentrations. Adding 10% carbon black improved the tensile strength (from 5.1 MPa to 6.8 MPa), indicating enhanced structural reinforcement. However, at 5%, it slightly decreases, suggesting that this concentration is not optimal for tensile strength. Elongation decreases consistently with increasing carbon black content, reflecting reduced flexibility. The contact angle increases with increasing carbon black content, indicating decreased hydrophilicity (greater water resistance). There was no data for brittleness at 15%, which suggests that this concentration results in films that are too brittle to test, limiting their practical application. The addition of 5% and 10% rice straw improved the tensile strength, with a significant increase of 10% (12.5 MPa). These findings suggest that rice straw effectively reinforces the structure of the film. While the tensile strength increased, elongation improved only at 10% rice straw, indicating a better balance of strength and flexibility at this concentration. The contact angle increases slightly to 5% but decreases significantly to 10%, suggesting improved wettability (or hydrophilicity) at higher concentrations. Like carbon black, rice straw is brittle at 15%, preventing successful film formation.

The mechanical properties of biodegradable polymers based on CS and different PVA and S materials are presented in Table 3. The tensile strength increased from 5.1 MPa to 7.4 MPa, and the elongation at break increased from 10.5% to 13.7%, increasing the PVA content from 3 to 5% in the biodegradable polymers. The increase in tensile strength and elongation at break is due to the cross-linking of the OH groups of PVA with CS and S [44]. When the biodegradable polymer CS/PVA/S at a ratio of 1/3/6 was mixed with carbon black (M3), the tensile strength increased from 7.4 to 12.5 MPa, and the elongation at break decreased from 10.5% to 7.3%. as shown in Table 3. However, when the biodegradable polymer CS/PVA/S ratio was 1/5/4 mixed with rice straw (M4), the tensile strength increased from 5.1 to 6.8 MPa. The elongation at break increased from 13.7% to 17.5%. The increase in tensile strength was attributed to the increased content of SiO2 in the rice and wheat straw [47,48]. The hydrophilicity and hydrophobicity of the polymers can be indicated by the water contact angle, which provides information about the hydrophilicity of the dried films. The effects of rice straw and carbon black on the hydrophilicity of the biodegradable polymers based on CS and different ratios of PVA and S are presented in Table 3. The contact angle increased from 78.0 to 85.0 deg as the ratio of PVA increased from 3% to 5%. However, with the addition of carbon black to M1, the contact angle increased from 78.0 (M1) to 85.5 (M3). Rice straw decreased the contact angle of the biodegradable polymer from 85.0 (M2) to 71.8 (M4) deg. The hydrophilicity of polymers depends on different factors, including the type of polymer, additive, ingredient, and filler [49,50].

### 3.5. Biodegradability Properties of PE/S Blend Films

Biodegradability decreases as the carbon black content increases (45 to 52 days). This indicates that carbon black hinders the decomposition of the films, likely due to its hydrophobic nature and inert characteristics. The lack of data for the 15% sample supports the observation of brittleness and lack of film usability at this concentration. Unlike carbon black, 10% rice straw significantly accelerated degradation (41 days vs. 65 days for M2). These findings suggest that the organic nature of rice straw enhances the biodegradability of the films. A slight reduction in degradation time (64 days) demonstrated that a lower rice straw content did not significantly alter biodegradability. Carbon black moderately improved the tensile strength but reduced the elongation, making the films stiffer. Rice straw significantly improved the tensile strength and elongation, particularly at 10%. Rice straw significantly enhances biodegradability, whereas carbon black hinders it. The addition of 10% rice straw is ideal due to its superior tensile strength, elongation, and acceptable contact angle.10% rice straw is also optimal due to the reduced degradation time. Both additives cause brittleness at 15%, making them unsuitable for film formation. This highlights the importance of balancing additive content for practical applications.

The biodegradation of the blended polymers CS/PVA/S films and blended polymers mixed with each carbon black and rice straw mixture was studied by immersing the films in soil for 80 days, and the results are presented in Table 4. The results showed that the degradation of blended polymer films with 3% PVA (M1) was 90% in 45 days and increased to 65 days for biodegradable polymers containing 6% PVA (M2). The increase in degradation time of the biodegradable polymer is due to the crosslinking of PVA with both CS and S. The addition of carbon black and rice straw to the biodegradable polymer CS/PV/S decreases the degradation time, as shown in Table 4. However, the addition of rice straw decreased the degradation time by 36%, whereas the addition of carbon black decreased the degradation time by 15%. The decrease in the degradation time of biodegradable films is caused by the biological activity (of microorganisms) of S and rice straw [51,52,53].

**Figure 6:**
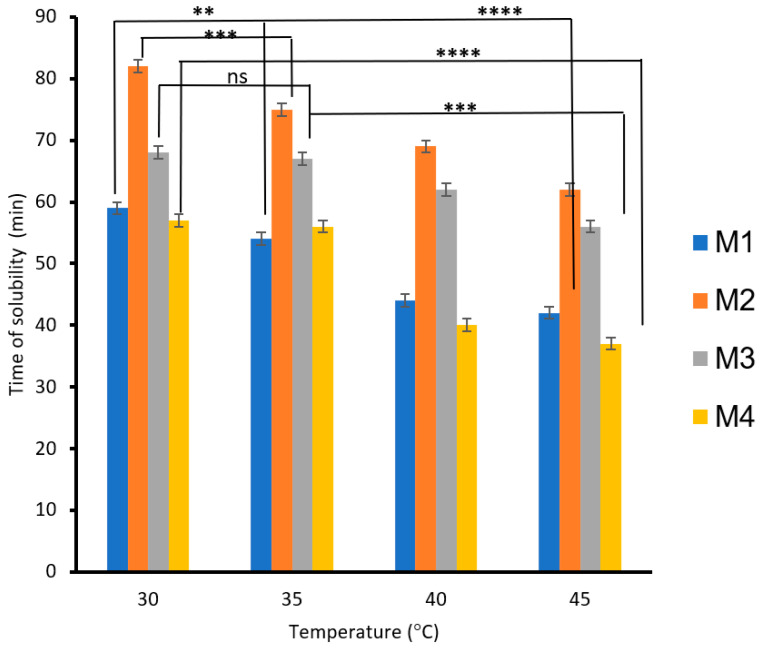
The effect of temperature on the biodegradability of films is due to the presence of carbon black and rice straw. A statistically significant difference is given as ****—*p* < 0.0001; ***—*p* < 0.001; **—*p* < 0.01 and no statistically significant difference—*p* > 0.05.

The effects of PVA, S, carbon black, and rice straw on the solubilities of the blended biodegradable polymer films, the films mixed with carbon black (M3), and the films mixed with rice straw as a function of time and temperature are shown in Figure 6. The results showed that the solubility of the biodegradable polymers increased as the temperature increased from 30 to 45 °C. The biodegradable polymer based on 3% PVA (M1) decreased the solubility time by 28%, whereas 6% PVA (M2) decreased the solubility time by 24%. However, adding carbon black to the biodegradable polymer M1 decreased the solubility time by 17% (M3), whereas adding rice straw to the biodegradable polymer M2 decreased the solubility time by 35% (M4). The decrease in solubility time is due to the role of rice mixed with CS/PVA/S, which increases the solubility of the biodegradable films with water more than the role of carbon black.

### 3.6. Scanning Electron Microscopy (SEM)

**Figure 7:**
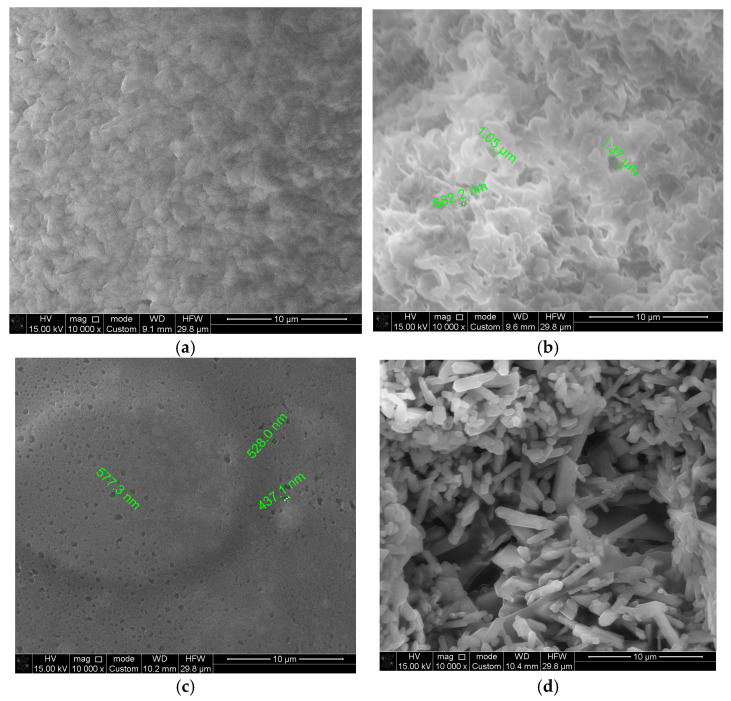
Scanning electron microscopy images of (**a**) CS/PV/S (M1); (**b**) CS/PV/S (M2); (**c**) M3 mixed with carbon black; and (**d**) M4 mixed with rice straw.

SEM images of the biodegradable polymer CS/PVA/S and biodegradable polymers mixed with carbon black and rice straw are shown in Figure 7. Figure 7a,b shows smooth and homogenous surfaces, indicating the miscibility of CS with PVA and S. However, the surface image of M1 (CS/PVA/S, 1/3/6) is rougher than that of M2 (CS/PVA/S, 1/5/4) because of the PVA content. Figure 7c shows that the fractured surface of the biodegradable polymers mixed with carbon black tends to coalesce due to the content of carbon black. Figure 7d shows the needles representing the rice straw mixed with biodegradable polymers. Small holes were observed on the sample surface (M1, M2, M3, and M4) due to S, CS, PVA, carbon black, and rice straw aggregation [54]. These results confirm the results of the water contact angle test for sample M4, which revealed that rice straw is a significant factor in controlling the hydrophilicity of the biodegradable material and increasing the biodegradability of the film through small holes.

## 4. Conclusions

Biodegradable polymers based on CS with different composition ratios of PVA and S were prepared by blending polymerization technique at ratios of 1/3/6 (wt. %) and 1/5/4 (wt. %), respectively. Carbon black and rice straw were mixed with biodegradable polymers to increase the biodegradability rate of the copolymer. The results revealed that the presence of rice straw in the blended polymer mixture increased the blended polymer’s thermal stability, mechanical properties, and biodegradability more than the presence of carbon black. Nonetheless, the CS/PVA/S mixture with a 1/5/4 ratio combined with rice straw presented superior mechanical qualities and a shorter deterioration time than the CS/PVA/S mixture with a 1/3/6 ratio blended with carbon black. Additionally, the biodegradability of the films increased by 36% with rice straw and by 15% with carbon black, highlighting the biological activity of these additives. The polymers also displayed increased solubility at elevated temperatures ranging from 30 °C to 45 °C, further broadening their potential applications.

## Figures and Tables

**Table 1:** The blend polymers are based on CS, PVA, and S with different ratios (wt.%).

Samples	CS	PVA	S
M1	1	3	6
M2	1	5	4

**Table 2:** Thermal properties of CS/PVA/S mixed with rice straw and carbon black.

Samples	Weight Lost %	Temperature, °C
M1	20	0–280
	41	280–340
	18	340–470
	25	470–500
M2	18	0–420
	75	420–480
M3	20	0–200
	25	200–380
	40	380–470
	15	470–600
M4	20	0–400
	60	400–500

**Table 3:** Mechanical properties of CS/PVA/S mixed with carbon black and rice straw.

Samples	Tensile Strength (MPa)	Elongation (%)	Contact Angle (deg)
M1	5.1	10.5	78.0
M2	7.4	13.7	85.0
M3	6.8	7.3	85.5
M4	12.5	17.5	71.8

**Table 4:** The effect of ground soil on the biodegradability of the films.

Samples	Time of Film Weight Loss Until 90% (Days)
M1	45
M2	65
M3	52
M4	41

## Data Availability

Data will be available upon request.

## Supplementary Materials

The following supporting information can be downloaded at: https://www.mdpi.com/article/10.3390/polym17040479/s1, Figure S1.
