# Supplementary material for: Obtaining and Characterization of Biodegradable Polymer Blends Based on Polyvinyl Alcohol, Starch, and Chitosan
Source: Polymers (Basel). 2025 Feb 12;17(4):479. doi: 10.3390/polym17040479 (PMC11860024; doi:10.3390/polym17040479)
Supplement: Supplementary file 1 [file polymers-17-00479-s001.zip › polymers-3424807-supplementary.pdf]

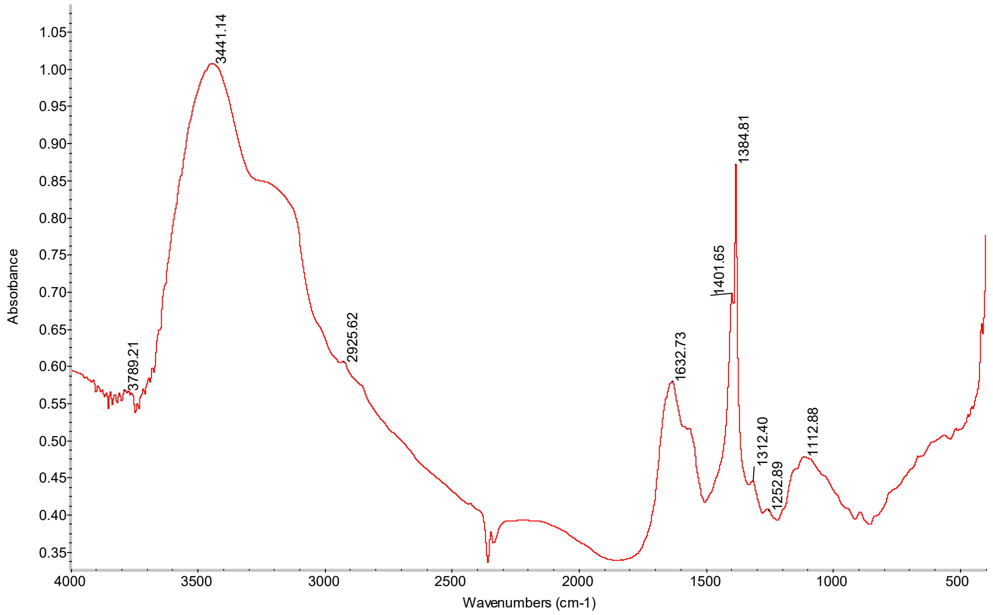

$$DA\ [\%] = \frac{A_{1655}}{A_{2870}} \times 100/1,33 \qquad (16)$$

= ((0.59/0.59) × 100)/1,33 = 75.2%

Fourier Transform Infrared ''FT-IR'' spectrophotometer, Thermo, Nexus, 670 FT-IR

Sigma-Aldrich.

3050 Spruce Street, Saint Louis, MO 63103, USA
Website: www.sigma-aldrich.com
Email USA: techserv@sial.com
Outside USA: eurtechserv@sial.com

Certificate of Analysis

**Product Name :**	**Chitosan low molecular weight**
**Product Number :**	**448869-250G**
**Batch Number :**	**0000400066**
Source Batch :	BCCL2629
CAS Number :	9012-76-4
MDL Number :	MFCD00161512
Quality Release Date :	02 JAN 2024

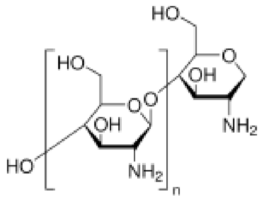

Test	Specification	Result
Appearance (Color)	Faint Beige to Beige	Faint Beige
Appearance (Form)<br>Powder and/Or Chips	Powder or Flakes	Powder
Assay<br>Deacetylation	≥ 75 %	79 %
Viscosity<br>c = 1%, 1% Acetic Acid	20 - 300 cps	172 counts/sec

Dr.Reinhold Schwenninger
Quality Assurance
Buchs,Switzerland
CH
